# Brillouin light scattering in niobium doped lead zirconate single crystal

**DOI:** 10.1038/s41598-022-17392-9

**Published:** 2022-07-29

**Authors:** D. Kajewski, S. H. Oh, J.-H. Ko, A. Majchrowski, A. Bussmann-Holder, R. Sitko, K. Roleder

**Affiliations:** 1 Institute of Physics, University of Silesia in Katowice, ul. 75 Pułku Piechoty 1, 41-500 Chorzow, Poland; 2 School of Nano Convergence Technology, Hallym University, Chuncheon, Gangwondo 24252 Republic of Korea; 3 Institute of Applied Physics, Military University of Technology, ul. gen. Sylwestra Kaliskiego 2, 00 -908 Warsaw, Poland; 4 Max-Planck-Institut für Festkörperforschung, Heisenbergstrasse 1, 70569 Stuttgart, Germany; 5 Institute of Chemistry, University of Silesia in Katowice, ul. Szkolna 9, 40-006 Katowice, Poland

**Keywords:** Ferroelectrics and multiferroics, Phase transitions and critical phenomena

## Abstract

Brillouin light scattering experiments were performed for lead zirconate single crystals doped with niobium. Special attention was paid to the elastic mode softening near phase transition temperatures. The results are compared with data obtained by Raman light scattering experiments. We observed that the interaction between acoustic and optic modes is responsible for symmetry breaking far above T_C_, leading to polar regions' appearance. No changes in the acoustic mode frequency and its damping are observed at T_C_, where ε(T) exhibits a maximum value. The absence of these changes and the central peak observed in Raman experiments suggest that the phase transition at T_C_ is mainly of the order–disorder type. The origin of other phase transitions is discussed as well.

## Introduction

Recently, the phase transitions of antiferroelectric PbZrO_3_ and related compounds have attracted increasing interest^1–6^. The search for new materials based on solid solutions of antiferroelectric (AFE) PbZrO_3_ or related perovskites, lead to novel compounds with enhanced piezoelectric properties. A good example is PbZr_1-x_Ti_x_O_3_ or PbZrO_3_ doped with Sn^7^. Such dopants result in complex phase diagrams and the appearance of intermediate phases not observed in pure PbZrO_3_ (PZO). The same applies to PZO doped with heterovalent ions, such as niobium ions^8–10^.

In Nb-doped PZO single crystals, symmetry breaking effect and two transient phases have already been observed above the Curie temperature (T_C_) and at temperatures well below T_C_^10^. Brillouin light scattering experiments in PZO and PbHfO_3_^11^, as well as tin-doped compounds^12,13^ have evidenced distinct acoustic mode anomalies associated with the phase transitions, substantial changes in the speed of sound propagation, and the formation of significant hypersonic damping^12,14^. This method is thus promising to analyse the aforementioned local symmetry breaking, both above T_C_ (pre-transitional effects) and below this transition (post-transitional effects), and determine its origin in PZO:Nb. The appearance of polar regions above T_C_ is associated with the coupling between transverse optical (TO) and acoustic mode (TA), which was theoretically predicted^15^. In that paper it was suggested that precursor dynamics are always present above T_C_ and are independent of the double-well potential's shape and depth. As an effect, polar clusters appear to overgrow on approaching T_C_ from the high-temperature side and reach several lattice constants' size at T_BH_ = T/T_C_ = 1.1 (with temperature given in Kelvins). However, this theory is about coupling the zone-centre TO phonon and zone-boundary TA phonon. This article aims to study the zone-centre LO phonons to check their behaviour connected with the precursor effects. At the same time, investigations of such elastic properties of PZO:Nb single crystal complement Raman light scattering already reported in^10^.

As suggested in Ref.^3^, based on the dielectric and optical properties measurements, the Raman spectroscopy proved the coexistence of phases above and below T_C_. The pure antiferroelectric state is observed only below 200 °C, whereas paraelectric-only behaviour starts above 320 °C, i.e. much higher than theoretically predicted T_BH_ mentioned above. As described in our recent papers 3, 9, and 10, this is caused by the existence of defects created by heterovalent dopant Nb^5+^. In general, in pure ABO_3_ perovskites, the paraelectric phase is realised only above T_BH_. Below this temperature, polar regions become stable, grow in size and start interacting, leading to phase transformation at T_C_. In PZO:Nb crystals, the paraelectric phase evolves above a temperature much higher than T_BH_. It suggests a temperature range above T_BH_ in which polar regions are unstable, i.e. their existence may fluctuate with a life-time long enough to detect them by Raman light scattering. In the PZO:Nb single crystal, for the two optical modes, the deviation of ω^2^(T) run from the linear dependence represents this scenario in the best way. Namely, the one mode changes its behaviour at T_BH_, and the second one at a temperature much higher than T_BH_^10^.

Doping PZO with Nb^5+^ unbalances charge neutrality of the lattice. To understand behaviour of the two modes mentioned above, especially the extension of the temperature range, in which polar regions exist in the paraelectric matrix, one must consider the interaction between lattice dynamics, defects and electrons introduced into crystal lattice by heterovalent ions^16^. Because of that, in the Raman spectra of PZO:Nb the so-called central peak (CP) was observed, and changes in its intensity were similar to changes of permittivity, with a sharp anomaly at T_C_. Such CP's feature is connected with some static or/and dynamic disorder in the lead sublattice^7^. Thus the CP existence is proof for the order–disorder transition at T_C_ associated with a relaxation process in this material.

In the study reported here, the Brillouin scattering results in a PbZrO_3_ single crystal doped with niobium in the amount of 1 mol% Nb_2_O_5_ are presented and compared to results obtained by the Raman scattering, with particular emphasis on the paraelectric phase. The Brillouin light scattering was applied to observe an acoustic mode in this matrial. This method has been used for a few decades to probe phase transition behaviors since the elastic properties of solids are sensitive to the changes of the order parameter. Especially, the elastic properties are strongly affected by the type of coupling between the order parameter (such as polarization) and the strain caused by acoustic waves. There have been numerous review papers on this subject, such as^17–22^. Therfore we believe that this investigation would prove the validity of the mentioned above coupling.

## Results and discussion

**Figure 1:**
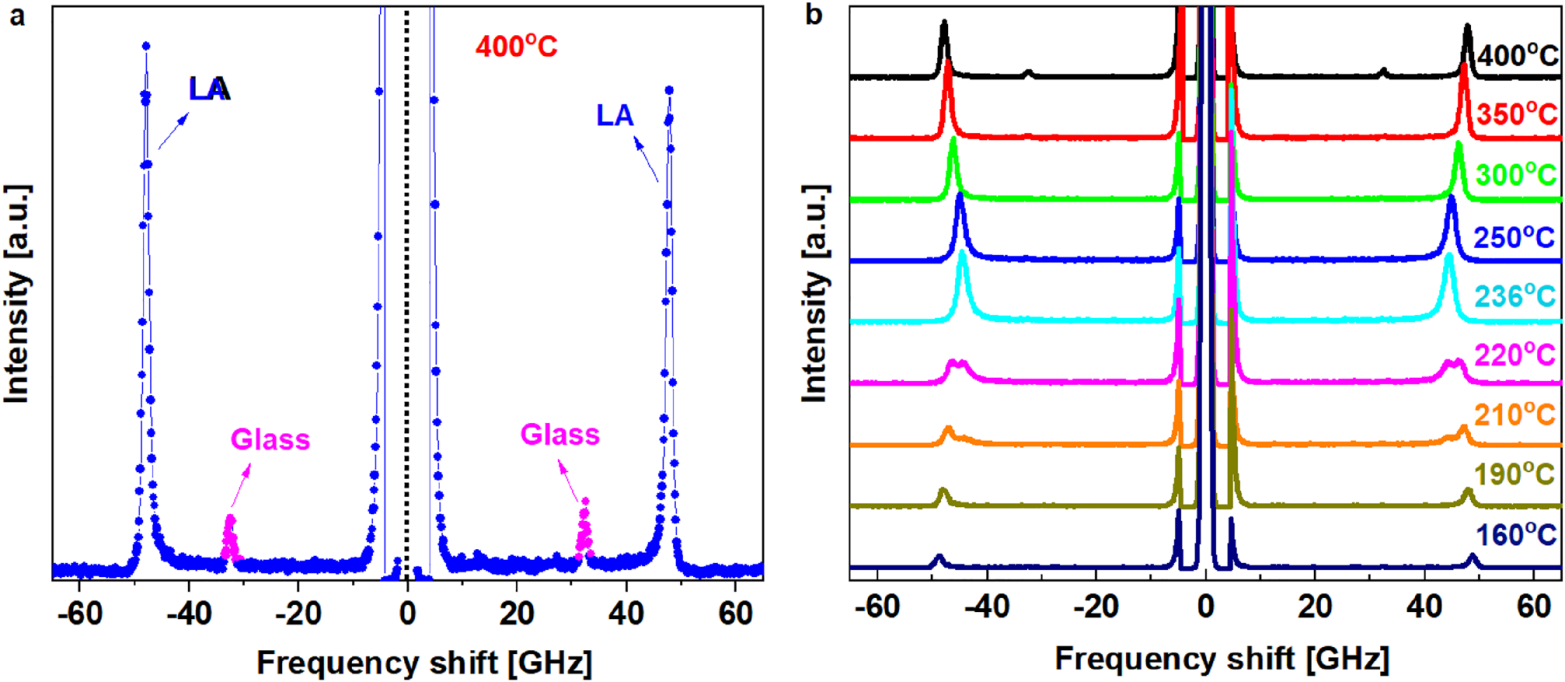
(**a**) Brillouin spectrum of PZO:Nb single crystal at 400 °C. The "Glass" denotes the LA mode coming from the glass substrate, which supports the thin PZO:Nb crystal; (**b**) Temperature dependence of the Brillouin spectrum for PZO:Nb. Splitting of the LA mode (LA1 and LA2 further in the text) is observed in the range 227–203 °C. In the (**b**) it is cleraly visible for temperatures 220 °C and 210 °C.

Figure 1 shows examples of the Brillouin scattering spectra obtained at different temperatures in the cooling process and the frequency range of ± 75 GHz. In Fig. 1a, two modes are clearly visible. The stronger one is the longitudinal acoustic (LA) mode of PZO:Nb propagating along the [100] direction, while the weaker one stems from the glass plate, which holds the sample in the heating stage. The signal observed in the range of ± 10 GHz, which comes from the Rayleigh peak, was cut off. In Fig. 1b, only one Brillouin doublet of the LA mode coming from PZO:Nb can be observed down to about 227 °C. Below this temperature, a splitting of the LA mode appears and persists up to about 203 °C. A further decrease in temperature leads to the disappearance of the "high-temperature branch". Unfortunately, no TA mode was observed in the whole temperature range. Most probably, it was connected with the experimental scattering geometry and the corresponding Brillouin selection rule^23^.

**Figure 2:**
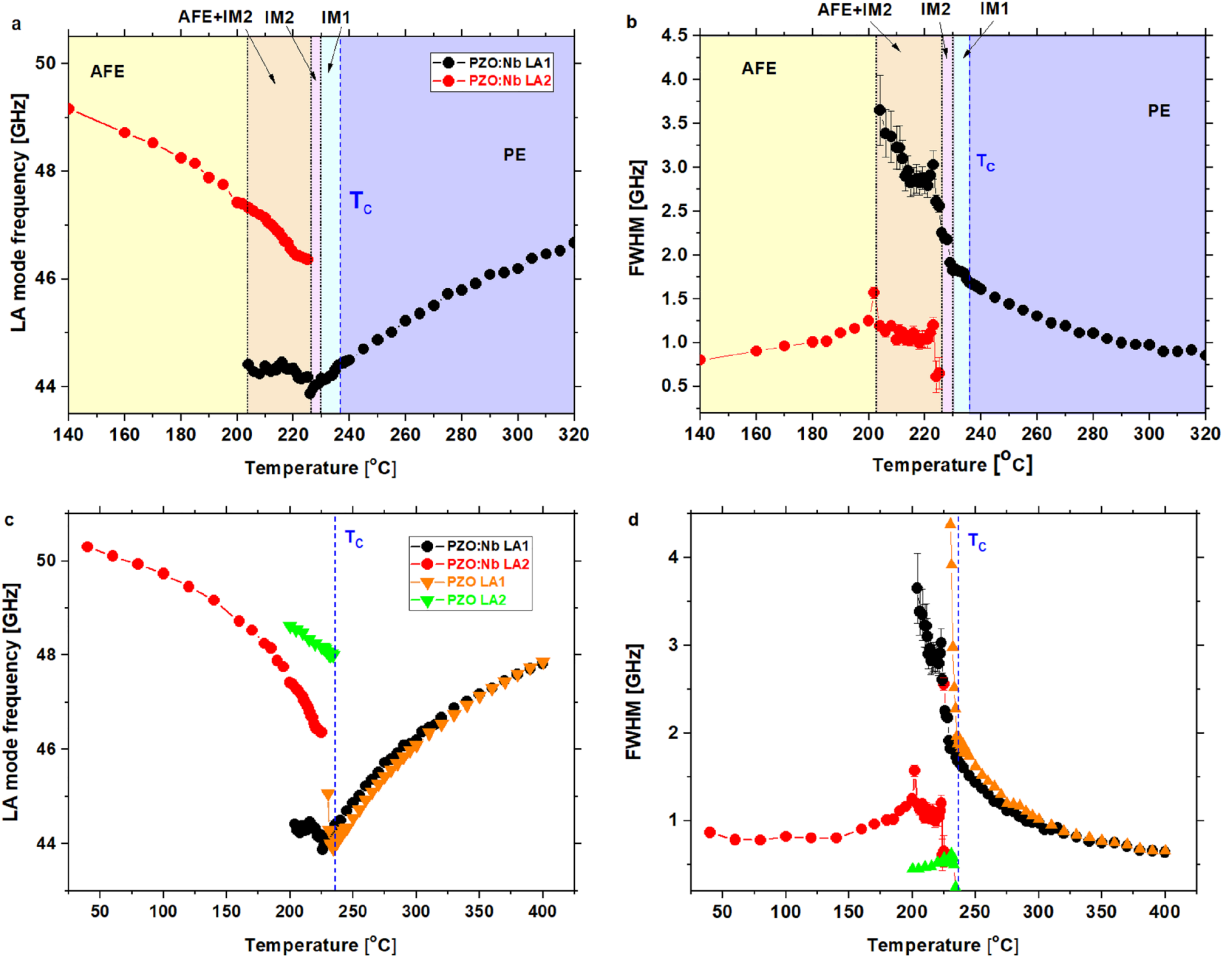
Temperature dependence of (**a**,**c**) the mode frequency and (**b**,**d**) the FWHM of the LA mode propagating along the [100] direction. The observed splitting of LA mode into LA1 and LA2 and their coexistence in PZO:Nb is similar to that observed in the intermediate phase of pure PZO.

Figure 2 shows the LA mode frequency shift's temperature changes and the full width at half-maximum (FWHM). Apparently, upon cooling softening of the LA mode takes place (Fig. 2a). This behaviour is similar to that observed in pure PZO (Fig. 2b)^3^, PbHfO_3_^24^, PbHfO_3_ doped with tin^12^ and BaTiO_3_^3^. The LA mode frequency (ν_B_) changes in PZO:Nb follow those observed in PZO down to about 310 °C, while below that temperature the LA mode frequency is slightly higher for PZO:Nb.

**Figure 3:**
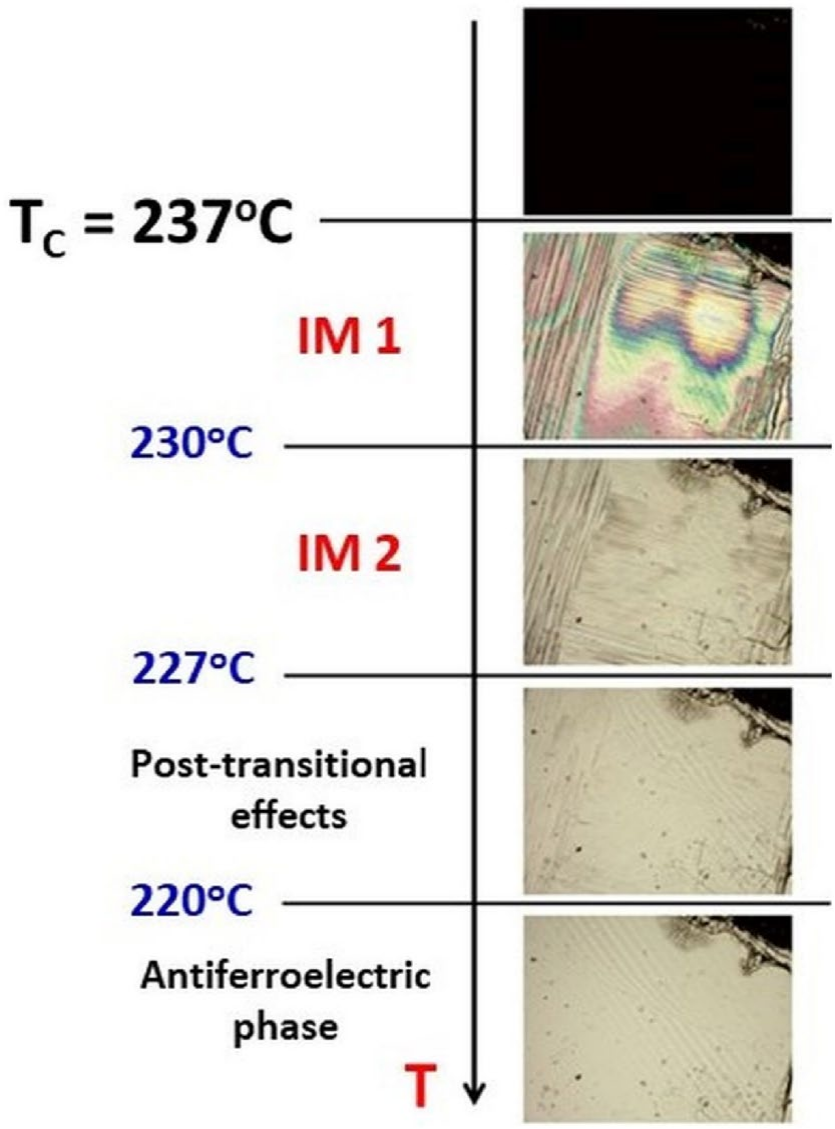
Images for PZO:1%Nb single crystal at selected temperatures under the polarised microscope. Domain structure disappears at T_C_, which correlates with a maximum permittivity^10^. IM1 and IM2 denote intermediate phases. Image size is c.a. 1.5 × 2 mm^2^.

A fascinating behaviour has been observed in the temperature range where phase transitions occur. All transition temperatures marked by vertical lines in Fig. 2a and c are taken from optical measurements presented in Fig. 3^10^.

The phase transition from the paraelectric state (PE) to the first intermediate phase (IM1) occurs at about T_C_(= 237 °C). The next phase transition occurs from IM1 to the second intermediate phase (IM2) at about 230 °C, and then the coexistence of phases is observed^10^ between 227 and 220 °C. It is clearly visible from Fig. 3 that the domain structure changes between IM2 and IM1 phase and then between between IM1 and coexistence of phase below 227 °C and at the end the domain structure is not observed below 220 °C. However, from observing the central peak in Raman scattering experiments, the coexistence of phases persists down to 200 °C. Correlating the above temperatures with possible anomalies in LA modes frequency, it is evident that only a slight aberration is visible at T_c_ (where the permittivity exhibits a maximum^10^), and further softening occurs with decreasing temperature (Fig. 2a). Also, a minimal change in damping takes place (Fig. 2c). A similar case was observed from the Brillouin scattering study of PbHf_0.7_Sn_0.3_O_3_^12^. In this crystal, the LA1 mode frequency did not show any minimum at the maximum permittivity temperature. The LA1 mode exhibits even further softening in the IM1 phase. It may suggest a lack of change in the crystal symmetry at T_C_, which stays in agreement with Raman scattering studies, in which no additional modes were observed^10^. Another slight anomaly is seen at IM1-IM2 phase transition (Fig. 2a), which is more pronounced in damping (Fig. 2c). Here, the LA1 mode frequency adopts a minimum value, and a splitting of this mode at 227 °C (Fig. 2a) occurs, which coincides with the IM2 macroscopic disappearance and the occurrence of the antiferroelectric state^10^. These peculiarities are associated with the increase in damping of the LA1 mode (Fig. 2c). Comparing these results with Raman scattering experiments suggests that the next acoustic LA2 mode is linked to the antiferroelectric state. At about 220 °C, an increase in damping in both LA1 and LA2 modes takes place together with a change in the Brillouin shift of the LA2 mode. This temperature corresponds to the disappearance of the IM2 phase as detected using a polarising microscope^10^ and Raman experiments of the Zr-O bending mode. With decreasing temperature, additional damping of the LA1 mode sets in, which is associated with relatively small frequency changes. At about 203 °C the LA1 mode disappears, which is consistent with the anomaly in Raman spectra.

Concerning undoped PZO (Fig. 2b and d), the two intermediate phases do not result from the antiferroelectric and paraelectric phase coexistence. However, a third intermediate phase (or post-transitional effects) emerges where two modes' coexistence is apparent. In both cases, the softening of the LA1 mode frequency and the increase in damping in the paraelectric phase are observed, caused by the appearance of polar regions^3^ stemming from the mode–mode coupling of the transverse optic TO mode and the corresponding transverse acoustic TA one^4,11^. As already mentioned, this effect has been observed in many other perovskites^3,11,24–26^. According to Bussmann-Holder et al., polar-nano-regions are related to the oxygen ion's nonlinear polarizability^27^. On the other hand, a kind of disorder in the oxygen octahedral tilts could be noted above T_C_^27^. Nb doping of PZO has a twofold effect. The transition metal ion is replaced by a higher valent one, and simultaneously the electrical misbalance is compensated by forming lead vacancies^8–10,28^. It is known that the lead-related vibrations are essential for the antiferrodistortive instability in PbHfO_3_ and PbZrO_3_^29^. Therefore, it can be concluded that doping PZO with Nb has influenced the formation of polar regions in the paraelectric phase. It is consistent with the results shown in Fig. 2b and d, i.e. it causes slightly weaker damping of the LA1 mode and a slight increase in the frequency of this mode below 310 °C, as compared to PZO. The increase in frequency could stem from the coupling of acoustic vibrations and oxygen octahedral tilt vibrations, as observed in the Raman spectra^10^. The latter are massively influenced by Nb ions' existence in the octahedral centre and the defects created in the lead sub-lattice^10^.

**Figure 4:**
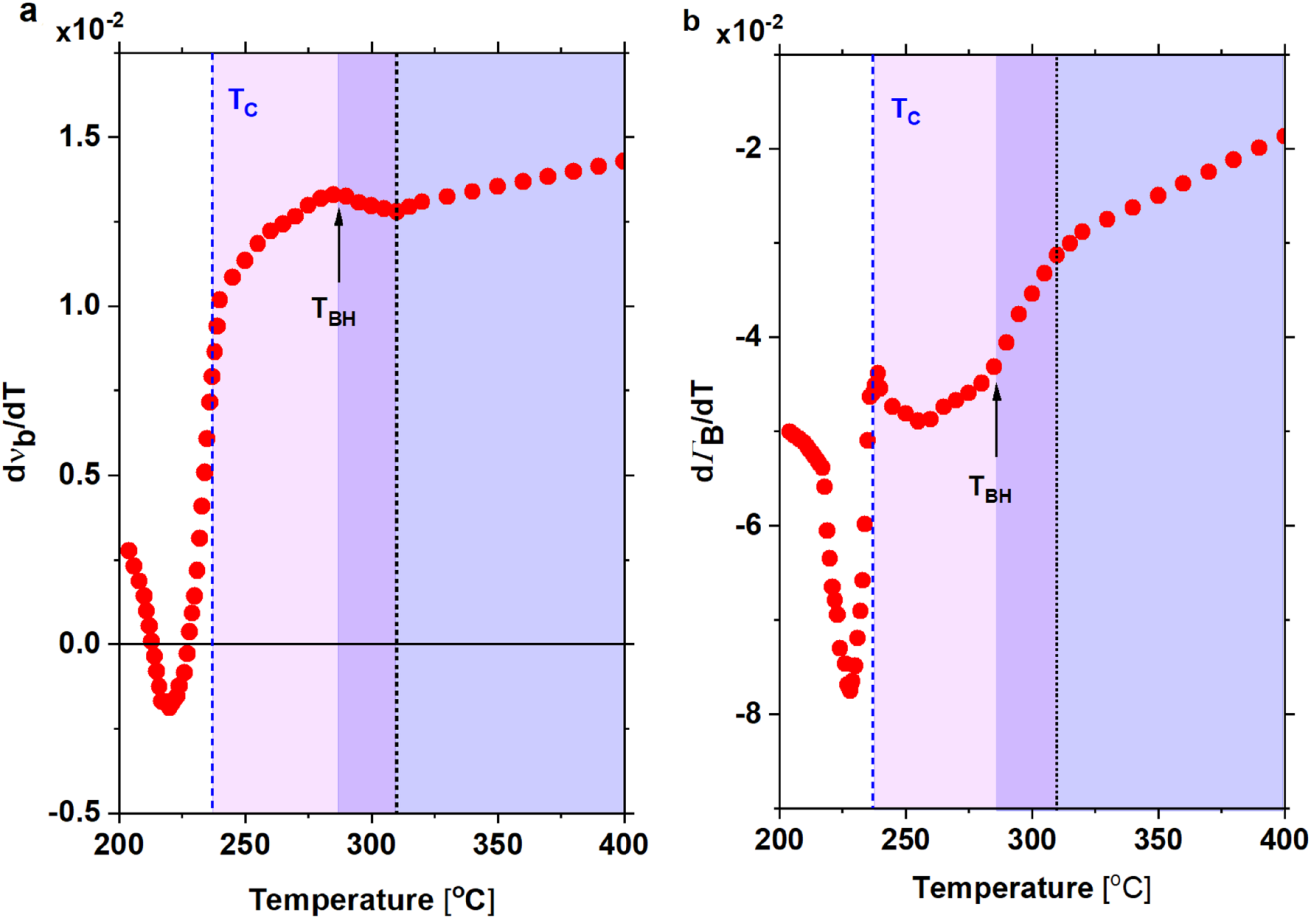
The first derivative of (**a**) frequency and (**b**) damping of the LA1 mode in PZO:Nb single crystal. Temperature changes of its frequency and damping coefficient above T_C_ reflect the temperature changes in the strength and damping of the central peak (CP) observed by Raman light scattering^10^. Anomalies of these values are visible at 310 °C and T_BH_. Also, the relaxor dumping changes between T_BH_ and T_C_, as reported in^10^, are divided in 255 °C into two parts, as the vibration damping of the LA1 mode.

So that to relate better T_C_ with the Brillouin light scattering results, the derivatives of frequency shift and damping for temperature have been assigned (Fig. 4). This approach has been used to obtain characteristic temperatures of relaxors and ferroelectrics^30^.

Indeed, the speed of changes in frequency and damping well corresponds to transition at T_C_. Also, an anomaly near 310 °C (marked with a slide line in Fig. 4) agrees with the temperature where polar nano regions, which come from defects, appear on cooling^10^. The next anomaly on cooling occurs at the Bussmann-Holder temperature T_BH_ = 1.1 ⋅ T_C_ equal to about 288 °C (for calculations, temperatures T_BH_ and T_C_ have to be taken in Kelvins). This anomaly is due to the appearance of polar nanoregions (precursors). Interestingly, the temperature range between T_BH_ and T_C_ is divided into two regions (Fig. 4b). In the first one, a decrease in the speed of changes in damping could be observed down to about 255 °C, and in the second region, the rate of changes in damping increases when temperature decreases. Such a feature could also be observed in crystals obtained in different crystal growth procedures on their piezoelectric activity above T_C_^9^. The first derivative of frequency for temperature takes zero at the temperature at which the LA2 mode appears. Its minimum occurs at a temperature where the disappearance of transient effects using a polarising microscope was observed (Fig. 3). The minimum damping derivative occurs strictly at a temperature where the IM2 phase disappears. The two derivatives' temperature changes also indicate that no extra heating of the sample is caused by laser light during measurements. Moreover, the minimum of LA1 mode and the LA2 mode's appearance does not occur in T_C_. This unexpected result was not observed in pure PZO crystal.

It should be noted that, once polar clusters appear, they enable the coupling to the elastic waves via the electrostrictive effect, i.e. the coupling between the squared polarisation and the strain caused by the longitudinal acoustic mode^18^. However, if the polar clusters are long-lived, we can expect local piezoelectric coupling in the clusters, where the cubic symmetry is locally broken^30^. When a polarisation occurs due to the acoustic strain field's action, it responds to and reacts to it^18^. This response is usually described as a relaxation process, and τ_LA_ is the relaxation time of this process. Since this process accompanies an energy exchange between the acoustic waves and the relaxational degree of freedom of the polar clusters, the energy dissipation is reflected in the damping of the LA mode, which is related to the phonon lifetime. This relaxation time can be derived from the abnormal changes in the frequency *ν*_B_ and the damping Γ_B_ of a LA mode through the following equation^31^

1 $$\tau_{LA} = \frac{{\Gamma_{B} - \Gamma_{\infty } }}{{2\pi \left( {\nu_{\infty }^{2} - \upsilon_{B}^{2} } \right)}}$$

where $\nu_{\infty }^{2}$ is unrelaxed squared Brillouin shift in the high-frequency limit, ${\Gamma }_{\infty }$ represents the high-frequency background damping which is not associated with the phase transition. Both quantities are derived from extrapolating toward the high-temperature region, where they exhibit nearly constant values.

**Figure 5:**
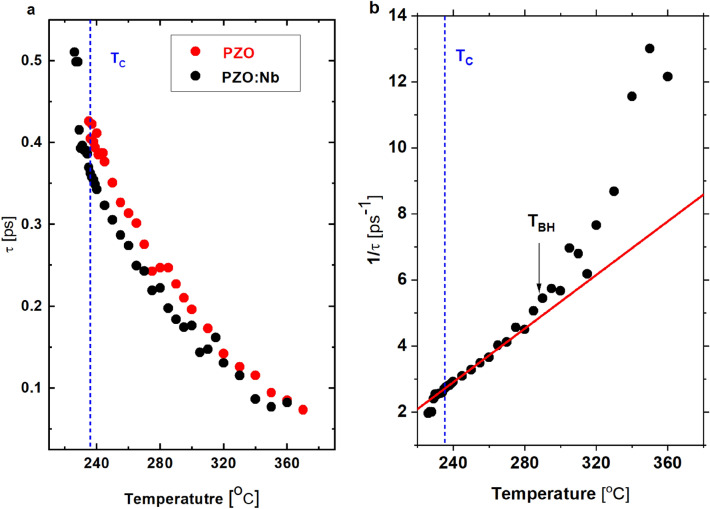
(**a**) Temperature dependence of relaxation time, and (**b**) inverse relaxation time derived from the abnormal changes in the LA1 mode frequency and its half-width.

Figure 5a shows the increase of relaxation time for LA1 mode with decreasing temperature for PZO and PZO:Nb. It signifies that the average volume of polar clusters increases and/or the interaction between the clusters is enhanced due to increased order parameter fluctuations near the phase transition temperature. Compared to pure PZO, doping with Nb ions causes a slight decrease in the relaxation time, suggesting a weakening in the interaction between polar nano regions or their smaller volume. It is consistent with the observation that the damping, represented by the half-width of the LA1 mode and is associated with the order-parameter fluctuations, becomes slightly smaller at temperatures below 310 °C compared to pure PZO. The overall changes in the mode frequency and damping in the PE phase are associated with polar clusters' squared local polarisation^18^. As described above, the Nb doping disturbs the oxygen octahedra tilts, creates Pb vacancies and, thus, is expected to be responsible for more negligible polar activity in the PE phase.

The inverse of the relaxation time is shown in Fig. 5b. Linear behaviour could be observed just below 280 °C, while T_BH_ = 288 °C for Nb-doped PZO. Such behaviour can be described using the following equation, which was initially suggested for the critical slowing down process in order–disorder systems^25^.

2 $$\frac{1}{{\pi \tau_{LA} }} = \frac{1}{{\pi \tau_{0} }}\frac{{T - T_{0} }}{{T_{0} }}$$

In this equation, T_0_ and τ_0_ are fitting parameters. The solid line in Fig. 5b denotes the best-fitted results, from which T_0_ = 168 °C and τ_0_ = 0.146 ps were confirmed. This kind of slowing-down behaviour was also observed in other systems, such as BaTiO_3_^25^, Pb(Sc_1/2_Ta_1/2_)O_3_^32^ and Ba_2_NaNb_5_O_15_^33^, which exhibit order–disorder behaviours, at least, partially.

The temperature dependence of the relaxation time and previous results of Raman scattering experiments^10^, i.e. the existence and behaviour of the central peak, lead to the conclusion that polar regions' presence must be correlated with Pb vibrations. Moreover, it is independent of the dopant ions and the defects generated by them. It also demonstrates the critical role of the coupling of the optic and acoustic zone-center vibrations. The linear behaviour of the inverse relaxation time near T_C_ is observed for perovskites, such as BaTiO_3_ and PbHfO_3_^25^. Hence, we state that phase transition's order–disorder nature also takes play in the PZO:Nb.

## Experimental

High-temperature solution growth of lead zirconate was chosen for the fabrication of single crystals since the compound melts incongruently at 1843 K^34^. Therefore, spontaneous crystallisation was achieved from self-flux containing Pb_3_O_4_ enriched with B_2_O_3_. The addition of boron oxide decreases the evaporation of lead oxide and does not cause any incorporation of boron atoms into PZO:Nb crystals. The exact composition of the starting melt was taken after Ref.^27^ as 2.4 mol% of PbZrO_3_:Nb^5+^ (1 at. %), 77 mol% of PbO (re-counted to Pb_3_O_4_) and 20.6 mol% of B_2_O_3_ (140 g in total). The use of Pb_3_O_4_ causes a significant improvement in the quality of PZO:Nb single crystals. As-grown crystals were fully transparent and colourless, whereas the use of PbO as a solvent lead to the crystallisation of greyish samples. The crystallisation was carried out in a platinum crucible covered with a platinum lid to reduce lead oxide evaporation. In the first step of crystallisation, the melt was soaked at 1300 K for 24 h to ensure the components' complete dissolution. Spontaneous crystallisation occurred upon cooling down to 1120 K, at the rate of 3.5 K/h. At this temperature, the melt was decanted, and PZO:Nb single crystals that had grown on the crucible walls were cooled to room temperature at the rate of 10 K/h. To remove the residues of solidified melt, as-grown PZO:Nb single crystals were etched in a hot water solution of acetic acid.

The chemical characterisation was performed employing an energy-dispersive X-ray fluorescence (EDXRF) spectrometer. The details of the procedure can be found elsewhere^10^. Chemical analysis revealed PbO excess of 2.15 ± 0.089 mol%, which might arise from the excess of Pb in the flux needed to prevent Pb vacancy formation at high temperatures. The actual amount of Nb_2_O_5_ was determined by the XRF technique, and was found to be 0.0772 ± 0.0037 mol% (0.0311 ± 0.0015 at.% of Nb), which is much smaller than the nominal concentration. This is related to the fact that Nb causes the creation of defects (two niobium ions could create one lead vacancy) and charge compensation through Pb vacancies^7^.

Brillouin spectra of PZO:Nb single crystals were acquired by using conventional tandem multi-pass Fabry–Perot interferometer (TFP-2, JRS Co.). TFP-2 utilizes the polarization state combined with the quarter wave retardation to remove any interferometric coupling and cross-talk resulting in high contrast ratio of 10^15^. Because of this modification, it has some limitations such as only vertically-polarized light onto the input pinhole can be analyzed. The sample size was approximately 3 × 1 × 0.1 mm^3^. The [100]-oriented sample was placed into a compact cryostat (THMS-600, Linkam). A modified microscope (BH-2, Olympus), in which a small prism was inserted to redirect the probe light to the objective lens, was used for backscattering experiments. A diode-pumped solid-state single-mode laser (Excelsior 532–300, Spectra Physics) at the wavelength of 532 nm and of a power ~ 10 mW was adopted to excite the crystal. A conventional photon-counting system combined with a multichannel analyzer (1024 channels) was utilized to detect and average the signal. The mirror spacing was 2 mm, and thus free spectral range was 75 GHz for measuring the Brillouin spectrum. The details of the experimental setup can be found elsewhere^35,36^. The Brillouin spectra were fitted by using a superposition of two kinds of response functions consisting of one (or two) damped harmonic oscillator(s) for the LA mode(s) and a single Debye relaxator for the CP.

## Conclusions

Single crystals PbZrO_3_ doped with niobium were studied using Brillouin light scattering spectroscopy. Special attention was paid to temperature dependences of the LA mode frequencies propagating along the [100] direction. The results lead to the following conclusions:

1. The appearance of two intermediate phases and the broad coexistence temperature range of a second intermediate phase with the low-temperature antiferroelectric phase were observed. The obtained results, combined with Raman light scattering investigations, show that the phase transitions in PZO:Nb are characterised by the simultaneous softening of the zone-centre optical and zone boundary acoustic modes.
2. Interestingly, no drastic change in the LA1 mode frequency and the damping was observed at T_C_, where ε(T) exhibits maximal value. It suggests no modifications of the crystal symmetry at T_C._ Slight changes for that mode at T_C_ and the temperature behaviour of the central peak observed in Raman scattering investigations mean that the phase transition from the PE to IM2 phase is mainly connected with the order–disorder transition mechanism.
3. The decrease in LA mode frequency and the simultaneous increase in the damping when approaching T_C_, must be attributed to polar regions' existence, which caused the mode softening and increasing damping on coming to the phase transition temperature via electrostrictive coupling.
4. Due to the interaction between the acoustic and optical zone-centre modes, the relaxation of the polar regions most likely takes place through the flexoelectric coupling, as considered in^5,37^.
5. The results demonstrate that T_BH_ does not depend on the existence of defects introduced by doping. It is universal to oxide perovskites and connected with a coupling between the zone-boundary acoustic and zone-centre optic modes.

## Data Availability

The datasets generated and analysed during the current study are available from the corresponding author on reasonable request.
